# From Pulmonary Tuberculosis to Antineutrophil Cytoplasmic Antibody (ANCA) Seroconversion: A Case of Rapidly Progressive Glomerulonephritis

**DOI:** 10.7759/cureus.103961

**Published:** 2026-02-20

**Authors:** Omar A AlShammari, Ibrahim Abuqurayn, Iffat kiran, Abdullah Almansour, Mohammad Almarzoqi, Bander Alazmi, Shifa Bilal Delvi, Abdulrahim Comert, Nada Abotouk, Raghad Bokhari, Basim A Amatouq, Abdulrahman A Almane

**Affiliations:** 1 Nephrology, King Fahad Medical City, Riyadh, SAU; 2 Internal Medicine, Alfaisal University College of Medicine, Riyadh, SAU; 3 Internal Medicine, King Fahad Medical City, Riyadh, SAU; 4 Pathology and Clinical Laboratory Medicine, King Fahad Medical City, Riyadh, SAU; 5 Infectious Diseases, King Fahad Medical City, Riyadh, SAU

**Keywords:** acute kidney injury, anca-associated vasculitis, kidney disease, rapidly progressive glomerulonephritis (rpgn), tuberculosis

## Abstract

Antineutrophil cytoplasmic antibody (ANCA)-associated vasculitis is a small-vessel inflammatory disorder that can lead to rapidly progressive glomerulonephritis. Although tuberculosis has been linked to ANCA positivity, the development of biopsy-confirmed immune-mediated kidney disease during anti-tuberculosis therapy is rare. Herein, we describe a rare presentation of delayed ANCA seroconversion associated with rapidly progressive glomerulonephritis in a patient undergoing pulmonary tuberculosis treatment. We report the clinical course, laboratory findings, histopathology, and management of the case. A 49-year-old man with pulmonary tuberculosis initially presented with mild renal impairment and negative autoimmune serology and was treated with standard anti-tuberculosis therapy. Several months later, he developed recurrent hemoptysis accompanied by rapidly worsening kidney function and active urinary sediment. Repeat serological evaluation demonstrated a new cytoplasmic staining pattern on indirect immunofluorescence for antineutrophil cytoplasmic antibodies, consistent with proteinase 3 antibody positivity on antigen-specific immunoassay. Renal biopsy revealed pauci-immune crescentic glomerulonephritis, confirming ANCA-associated rapidly progressive glomerulonephritis. Following a multidisciplinary discussion, immunosuppressive therapy was initiated, and anti-tuberculosis treatment was continued. Despite the therapy, the patient remained dialysis dependent. In summary, delayed ANCA seroconversion may occur during anti-tuberculosis therapy and may be associated with severe immune-mediated renal disease. Kidney biopsy is essential when unexplained kidney deterioration develops during treatment. In selected patients with severe organ involvement, immunosuppressive therapy may be required despite active infection, following careful multidisciplinary evaluation.

## Introduction

Antineutrophil cytoplasmic antibody (ANCA)-associated vasculitis is a heterogeneous group of autoimmune diseases that involve inflammatory processes affecting small-to-medium-sized blood vessels, resulting in various systemic and/or organ-specific manifestations [1]. The main types of ANCA include granulomatosis with polyangiitis (GPA), microscopic polyangiitis (MPA), and eosinophilic granulomatosis with polyangiitis (EGPA). GPA mostly results from anti-PR3 antibodies that cause persistent inflammation in the upper/lower respiratory system and the renal glomerulus, resulting in various concurrent symptoms [2,3].

MPA occurs due to anti-myeloperoxidase (MPO) autoantibody reactions in 70% of cases, involving the kidneys and rarely affecting the lungs, leading to a rapid deterioration in kidney function, eventually leading to end-stage renal disease (ESRD) [2,3].

EGPA occurs due to two coexisting pathophysiological pathways: type 2 T helper lymphocytes (Th2 lymphocytes) and ANCA. Th2 lymphocytes release interleukin (IL)-5 and IL-13, leading to eosinophil activation, eventually leading to eosinophilia. This combination of pathways leads to systemic manifestations and renal dysfunction. However, it typically manifests asthmatic symptoms along with glomerulonephritis [2,4].

In ANCA vasculitis, indirect immunofluorescence is performed for all types of vasculitis to detect c-ANCA or p-ANCA. To further differentiate between these types, a tissue biopsy is performed. GPA kidney biopsy reveals vasculitis and necrotizing granuloma, which are key features. In contrast, in MPA renal biopsy, the sample shows vasculitis with the absence of granulomas, except for a renal biopsy. Furthermore, MPA with positive p-ANCA and anti-MPO results is reported in approximately 70% of the cases; 20% of the cases are PR3-ANCA-positive, and 10% of the cases are ANCA-negative. Over 90% of GPA cases are positive for c-ANCA, and only 10% are positive for p-ANCA [4].

For EGPA, 35% of the cases are MPO-positive, while 5% are anti-PR3; however, 60% of the cases are ANCA-negative. Another finding that supports the diagnosis of EGPA is eosinophilia in the peripheral blood smear of affected tissues [4].

ANCA vasculitis presents with constitutional symptoms such as fever, weight loss, and fatigue. It also presents with single-and/or multiorgan involvement, such as respiratory involvement in GPA, kidney involvement in MPA, and nervous system involvement in EGPA [5].

Infectious agents can trigger and/or exacerbate vasculitis. Several viruses and bacteria, such as the hepatitis C virus, cytomegalovirus, Epstein-Barr virus, parvovirus, and *Staphylococcus aureus*, have been associated with vasculitis; however, the associative mechanisms remain unclear [6]. Moreover, certain medications trigger ANCA-associated vasculitis, which usually manifests as rapidly progressive glomerulonephritis (RPGN). Unlike other types of ANCA-associated RPGN, medication-induced ANCA has a shorter therapy induction period, and relapse is rare after discontinuation of the implicated agent. The most common drugs associated with ANCA vasculitis are hydralazine, propylthiouracil, methimazole, allopurinol, sulfasalazine, minocycline, penicillamine, rifampicin, aminoguanidine, sofosbuvir, and anti-tumor necrosis factor-alpha (TNF-α) [7]. Patients with tuberculosis are susceptible to ANCA vasculitis via multifactorial causative mechanisms [8]. Nonetheless, molecular mimicry between the effects of tuberculosis bacterial infection and the pathophysiology symptoms of ANCA plays a significant role in triggering the disease. One proposed mechanism suggests that cytokines released in tuberculosis infection cause the release of ANCA antigens, activating neutrophils, which in turn adhere to the vessel wall and release toxic oxygen radicals, resulting in the apoptosis and necrosis of the neutrophils and the adjacent vessel walls [9]. Diagnosing GPA and tuberculosis can be challenging, especially in tuberculosis-endemic areas, as both diseases have overlapping clinical features, such as cough, hemoptysis, fever, malaise, loss of appetite, and weight loss [10]. 

## Case presentation

We report the case of a 49-year-old man with a history of smoking who presented to the emergency department in March 2025 with a cough, weight loss, and night sweats. Polymerase chain reaction testing confirmed pulmonary tuberculosis, and he started on rifampin, isoniazid, pyrazinamide, and ethambutol (RIPE) therapy. He was referred to the nephrology department for abnormal renal function test results, which at that time showed serum creatinine between 150 and 209 µmol/L, along with urine microscopy showing a red blood cell count of 34 cells/high-power field and 24-hour proteinuria of 1.7-1.9 g/day. His autoimmune panel, including ANCA, antineutrophil cytoplasmic antibody (ANA), double-stranded DNA (dsDNA), and anti-glomerular basement membrane (GBM) antibodies, was negative, and complement levels were within the normal range (Table 1). A renal biopsy was performed, although the patient had initially declined to participate.

**Table 1:** Laboratory findings at initial presentation and during relapse ANCA: antineutrophil cytoplasmic antibody; ANA: antinuclear antibody; anti-GBM: anti-glomerular basement membrane antibody; dsDNA: double-stranded DNA; RBCs: red blood cells. Note: Reference ranges are laboratory standard adult values.

Parameter	March 2025	August 2025	Reference Range
Serum creatinine (µmol/L)	150	766	64–104
ANCA (c-ANCA) (U)	<20	73.58	<20
ANA	Negative	—	Negative
Anti-GBM antibody	Negative	—	Negative
Anti-dsDNA antibody	Negative	—	Negative
Urine RBCs (cells/high-power field)	34	1,683	0–2
24-hour urine protein (g/day)	1.9	—	<0.15

In the last week of August 2025, he developed worsening dyspnea, fever, recurrent hemoptysis, oliguria, and dark urine. Repeat evaluation revealed rapidly worsening kidney function, with creatinine rising from 209 µmol/L to 766 µmol/L, metabolic acidosis, hyperkalemia, and marked hematuria (1,683 red blood cells/µL). Repeat autoimmune testing demonstrated ANCA seroconversion (c-ANCA 73.58 U; normal <20 U) (Table 1).

**Figure 1:**
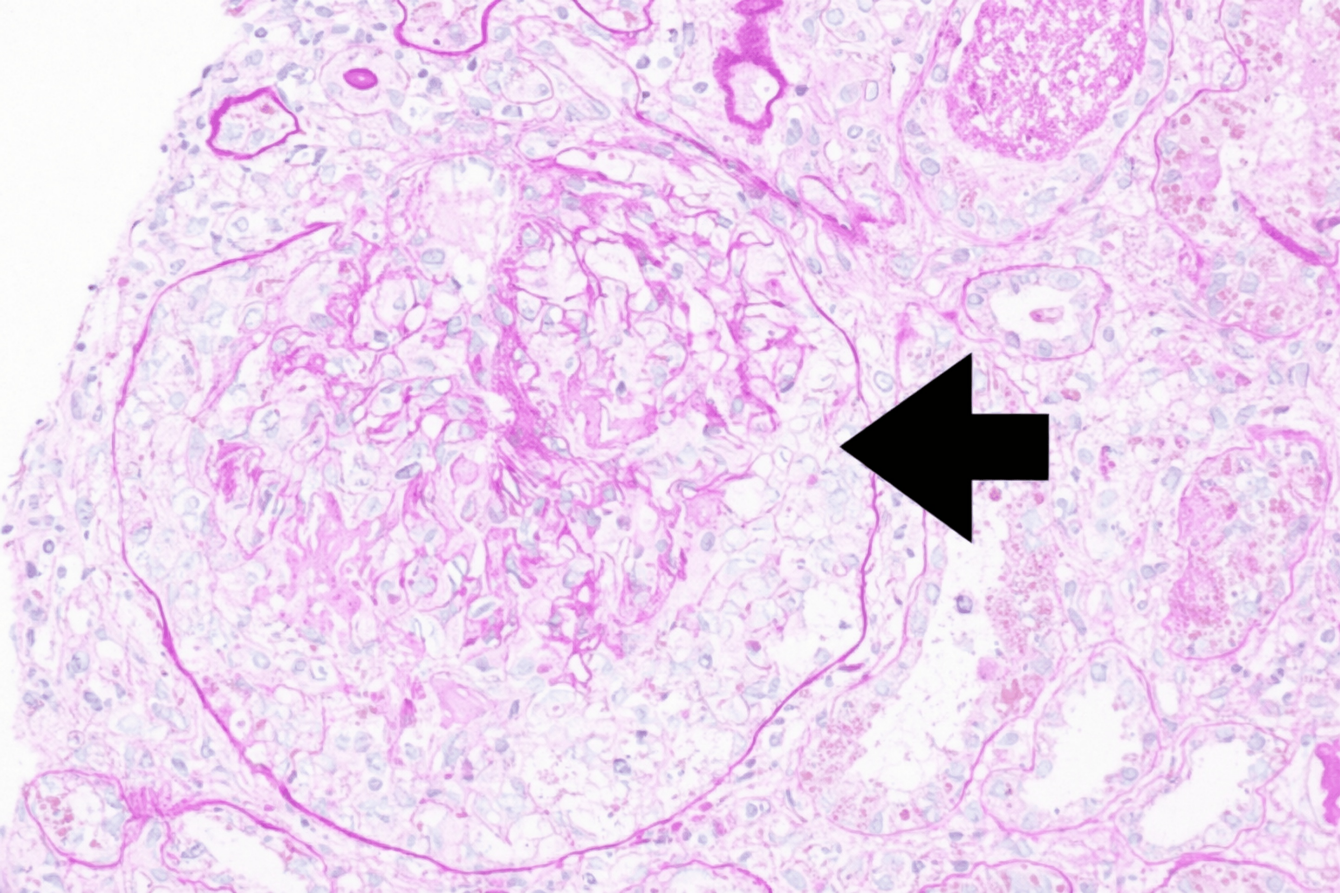
Kidney biopsy: Light microscopy (periodic acid–Schiff stain, ×400 magnification) showed a glomerulus with a cellular crescent (arrow).

**Figure 2:**
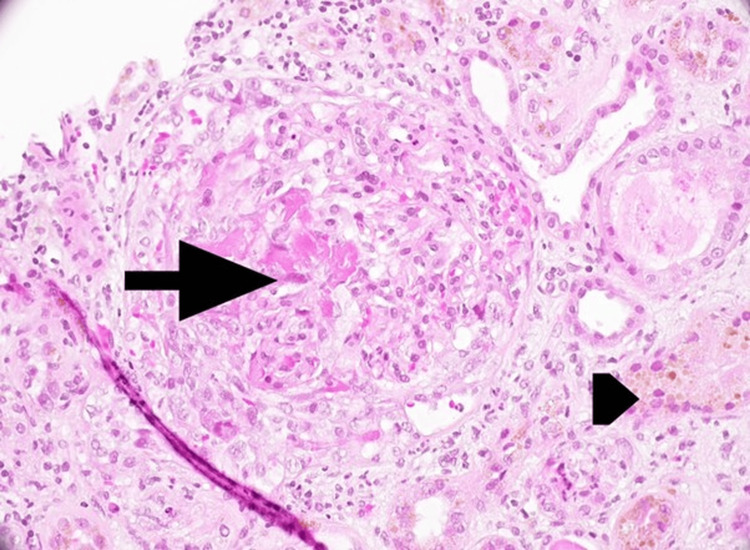
Kidney biopsy: Light microscopy (haematoxylin and eosin stain, ×400 magnification) demonstrated a glomerulus with a cellular crescent and segmental fibrinoid necrosis (arrow). The tubules exhibited pigment epithelial changes (arrowhead).

A renal biopsy performed in August revealed pauci-immune crescentic glomerulonephritis with seven cellular crescents, acute tubular injury, pigmented casts, and acute pyelonephritis, consistent with RPGN (Figures 1, 2). Additionally, the patient developed pulmonary hemorrhage during hospitalization. Given the ongoing anti-tuberculosis treatment, hemodialysis was initiated, followed by immunosuppressive therapy, after a multidisciplinary discussion with the infectious disease team. He received pulse methylprednisolone 500 mg intravenously for three days, plasma exchange with intravenous immunoglobulin (IVIG) for a total of seven sessions, and rituximab (1 g administered twice, two weeks apart), while RIPE therapy continued. Despite the therapy, the patient remained dialysis dependent.

Immunofluorescence showed no significant immune complex deposition. Electron microscopy did not demonstrate electron-dense deposits.

## Discussion

This case illustrates the complexity of diagnosing and managing renal disease in patients with active tuberculosis. At presentation, the patient had pulmonary tuberculosis with mild renal impairment and negative ANCA serology. Near the completion of anti-tuberculosis therapy, kidney function deteriorated rapidly, indicating the emergence of a distinct and aggressive pathological process.

Although delayed ANCA seroconversion during or after anti-tuberculosis treatment has been described earlier [10], progression to clinically significant glomerular disease remains uncommon. As shown in Table 2, only a few reported cases have documented biopsy-confirmed renal involvement, highlighting the rarity of this presentation.

**Table 2:** Summary of published case reports describing tuberculosis-associated with ANCA serology and glomerular disease, with emphasis on renal involvement, timing of ANCA detection, and immunosuppressive strategies. Cases are ordered according to the presence and severity of kidney involvement [11, 12, 13]. Reports without renal disease are excluded from this table. ANCA: antineutrophil cytoplasmic antibody; IV: intravenous; GN: glomerulonephritis; TB: tuberculosis; AKI: acute kidney injury; MPO-ANCA: myeloperoxidase-antineutrophil cytoplasmic antibody; MPGN: membranoproliferative glomerulonephritis; RIPE: rifampin, isoniazid, pyrazinamide, and ethambutol; eGFR: estimated glomerular filtration rate

Case	Timing of ANCA positivity	Immunosuppressive therapy	Renal involvement and outcome	Overall outcome
Oxley-Oxland et al. (2018) [11]	ANCA negative at presentation and on repeat testing	Anti-TB therapy (RIPE) plus IV methylprednisolone pulses, followed by oral prednisone taper and IV cyclophosphamide	Severe AKI; biopsy-proven pauci-immune crescentic GN (~50% crescents). Renal function recovered to near-normal	Completed anti-TB therapy; immunosuppression stopped after induction; sustained renal and clinical remission
O'Brien et al. (2021) [12]	ANCA seroconversion during anti-TB therapy (~2 weeks)	IV methylprednisolone, oral steroid taper over 4 months, mycophenolate mofetil; anti-TB therapy continued	Crescentic GN with severe AKI. eGFR improved from 8 to ~50 mL/min/1.73 m²	Clinical improvement after immunosuppression
Murakami et al. (2024) [13]	MPO-ANCA positive at diagnosis of GN in latent TB	No immunosuppressive therapy	MPGN with gradual improvement in kidney function and proteinuria	Favorable outcome with conservative management

Although there have been many cases of circulating ANCA in patients with tuberculosis [14,15], several features distinguish this case from previous reports, including initial ANCA negativity, rapid progression to dialysis-dependent kidney failure, and concurrent pulmonary hemorrhage. The coexistence of these findings presents a major therapeutic challenge, as conventional vasculitis treatment frameworks provide limited guidance in the presence of active infection.

In this context, a renal biopsy is essential for establishing a diagnosis and guiding management. The severity of renal injury and life-threatening pulmonary involvement justified the cautious initiation of immunosuppressive therapy despite the ongoing tuberculosis treatment. This decision was supported by a close multidisciplinary collaboration and continuous reassessment. Overall, this case underscores the need for individualized treatment strategies when immune-mediated renal disease complicates tuberculosis.

## Conclusions

Tuberculosis may be associated with delayed ANCA seroconversion and subsequent immune-mediated renal injury. Immune reconstitution or paradoxical inflammatory responses during anti-tuberculosis therapy may contribute to this phenomenon. Kidney biopsy remains central to differentiating ANCA-associated glomerulonephritis from infection-related renal pathologies. In the presence of severe renal or pulmonary involvement, carefully selected immunosuppressive therapy may be justified despite active infection, following a multidisciplinary evaluation. ANCA testing should be interpreted within the broader clinical and histopathological context.
